# Differences in heat tolerance, water use efficiency and growth among Douglas-fir families and varieties evidenced by GWAS and common garden studies

**DOI:** 10.1093/aobpla/plad008

**Published:** 2023-03-01

**Authors:** Samuel Compton, Charles Stackpole, Aalap Dixit, Manoj K Sekhwal, Thomas Kolb, Amanda R De la Torre

**Affiliations:** School of Forestry, Northern Arizona University, 200 E. Pine Knoll, AZ 86011, USA; School of Earth and Sustainability, Northern Arizona University, 624 S Knoles Dr Flagstaff, AZ 86011, USA; Department of Forestry, New Mexico Highlands University, Las Vegas, NM 87701, USA; School of Forestry, Northern Arizona University, 200 E. Pine Knoll, AZ 86011, USA; School of Forestry, Northern Arizona University, 200 E. Pine Knoll, AZ 86011, USA; School of Forestry, Northern Arizona University, 200 E. Pine Knoll, AZ 86011, USA

**Keywords:** Water use efficiency, carbon isotope discrimination, heat tolerance, electrolytic leakage, Douglas-fir, GWAS, hybridization

## Abstract

Severe and frequent heat and drought events challenge the survival and development of long-generation trees. In this study, we investigated the genomic basis of heat tolerance, water use efficiency and growth by performing genome-wide association studies in coastal Douglas-fir (*Pseudotsuga menziesii*) and intervarietal (*menziesii* × *glauca*) hybrid seedlings. GWAS results identified 32 candidate genes involved in primary and secondary metabolism, abiotic stress and signaling, among other functions. Water use efficiency (inferred from carbon isotope discrimination), photosynthetic capacity (inferred from %N), height and heat tolerance (inferred from electrolyte leakage in a heat stress experiment) were significantly different among Douglas-fir families and varieties. High-elevation seed sources had increased water use efficiency, which could be a result of higher photosynthetic capacity. Similarly, families with greater heat tolerance also had higher water use efficiency and slower growth, suggesting a conservative growth strategy. Intervarietal hybrids showed increased heat tolerance (lower electrolyte leakage at 50 and 55 °C) and higher water use efficiency compared with coastal families, suggesting that hybridization might be a source of pre-adapted alleles to warming climates and should be considered for large-scale reforestation projects under increasingly arid conditions.

## Teaser

Warmer climates bring significant challenges to the survival of coastal Douglas-fir natural and managed populations due to their low tolerance to heat and low water use efficiency. Our study indicates that families from higher elevation and those having some mixed ancestry from the interior variety might be better adapted to future climate conditions.

## Introduction

High temperatures are an emerging, climate-driven stress that can cause numerous biochemical, physiological and morpho-anatomical changes in plants leading to stunted growth and development (Kotak *et al.* 2007; Wahid *et al.* 2007). Drought, caused by low soil water content and/or high evaporative demand, often accompanies high temperature and is one of the most important climatic stressors affecting growth, performance and survival of plant species (McDowell *et al.* 2008; Estravis-Barcala *et al.* 2020). When working in tandem, drought and heat stress have the potential to cause permanent physiological harm to plants (Shah and Paulsen 2003; Wang and Huang 2004). Climate change is predicted to cause more extreme temperatures and alter precipitation regimes, causing an increase in the severity and duration of heat stress and drought leading to reduced growth and increased tree mortality (Loarie *et al.* 2009; Trumbore *et al.* 2015). While coniferous gymnosperms are the dominant trees of global arid forests and are generally considered more heat and drought resistant than angiosperms, investigation of the genetics of heat and drought stress in gymnosperms has focused on only a few species and lags studies in angiosperms, especially crops (Moran *et al.* 2017). Understanding the genetics and physiology of heat and drought tolerance can help us better predict the effects of climate change on gymnosperms and develop management strategies that target the hardiest genotypes for tree improvement and reforestation.

The physiological and genomic basis of drought tolerance in conifers have been investigated via transcriptomic, genomic and/or common garden studies (reviewed by Moran *et al.* 2017). Common garden studies of commercial conifer species have provided information on the differential response to drought among populations or seed sources (Montwe *et al.* 2015; Bansal *et al.* 2016; Dixit *et al.* 2022). The recent development of sequencing technologies has facilitated investigation of genome-wide drought responses via gene expression analyses, thereby enabling the identification of candidate genes involved in stress responses (Behringer *et al.* 2015; Hess *et al.* 2016; Du *et al.* 2018; De María *et al.* 2020; Haas *et al.* 2021). Candidate genes identified by transcriptomic studies or other methods can be associated with specific drought-related traits through genome-wide association studies (Neale and Savolainen 2004; Cumbie *et al.* 2011). However, these methods have received less attention in conifers due to the difficulties in identifying large numbers of genes with limited number of reference genomes and incomplete annotations, and due to the extensive time required to obtain phenotypic data.

Douglas-fir (*Pseudotsuga menziesii,* Pinaceae family) is a long generation, mostly outcrossing, evergreen conifer species of great economic and ecological importance in North America (St. Clair *et al.* 2005). The species is categorized into three varieties based on their geographic location along the Pacific coast (coastal, var. *menziesii*), across the mountainous regions from the Rocky Mountains to Arizona (interior, var. *glauca*), or in the Sierra Madre Mountain ranges of Mexico (Mexican, var. *lindleyana*) (St. Clair *et al.* 2005). The coastal and interior varieties naturally hybridize in British Columbia (Canada), and the Washington Cascades (USA). Douglas-fir natural hybrids have been understudied, and their adaptation and stress response are unknown. Douglas-firs can grow under a variety of conditions, but the climate in which they are most found consists of warm, dry summers and cool, wet winters. Their natural species’ distribution range in North America spans from 19°N to 55°N degrees latitude and from sea level to over 3000 m of elevation (Hardin *et al.* 2001; Gould *et al.* 2011). Drought is present throughout a large part of the Douglas-fir’s geographic range, and climate change brings warmer temperatures on a global scale; as such, being able to identify which families and varieties of Douglas-fir are the most heat and drought tolerant is of key importance to foresters (Bansal *et al.* 2016).

Morphological, physiological and plastic responses to drought have been widely studied in Douglas-fir (Müller *et al.* 2012; Bansal *et al.*^2015^, ^2016^; Hess *et al.* 2016; Marias *et al.* 2016). In contrast, the genomics of heat tolerance and traits associated with drought tolerance have not been investigated in any variety or hybrids of the species. In addition to evaluating heat tolerance by cellular electrolyte leakage (Ruter 1993; Agarie *et al.* 1995), we measured seedling growth and the trait of carbon isotope discrimination, a time-integrated measure of photosynthetic water use efficiency (Farquhar *et al.* 1982). The lower carbon isotope discrimination of woody plants of dry habitats compared with plants of wet habitats (Diefendorf *et al.* 2010) suggests that water use efficiency is an important drought adaptation. These traits are relevant to conifer adaptation to arid conditions, as trade-offs between growth rate and water use efficiency have been reported in other conifers (Kerr *et al.* 2015; Dixit *et al.* 2022). The objectives of this study were to (a) investigate differences in water use efficiency, cellular heat tolerance and growth among families and varieties of Douglas-fir and (b) investigate the genomic basis (candidate genes, gene families and pathways) of the measured traits.

## Materials and Methods

### Greenhouse experimental design

**Figure 1.**
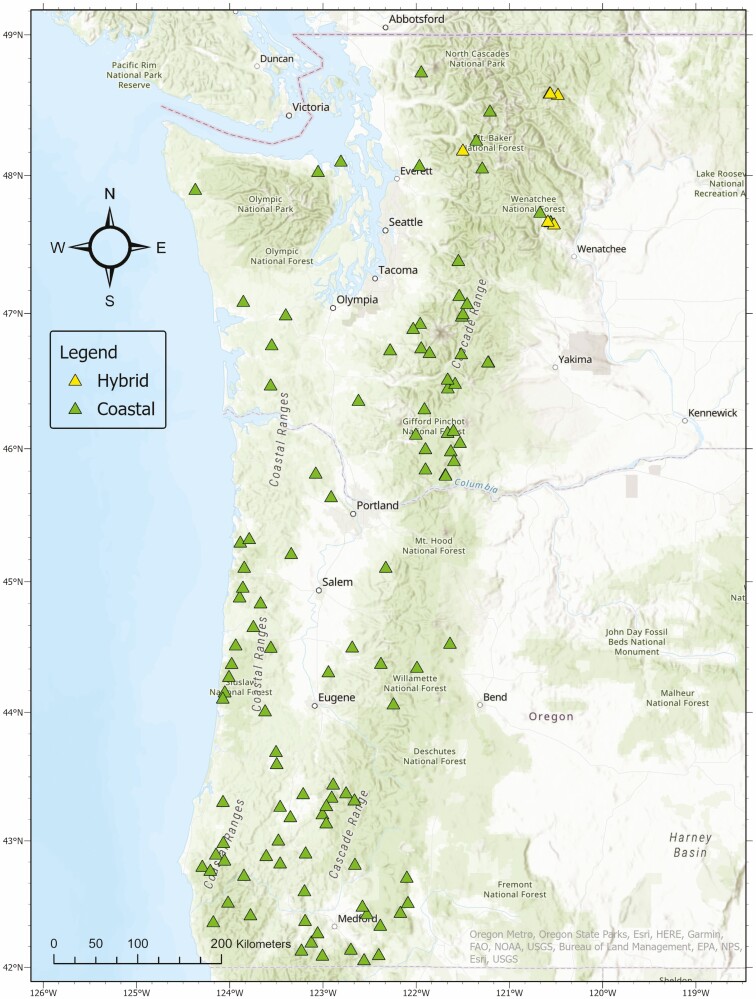
Geographic distribution of coastal and intervarietal hybrid Douglas-fir families included in this study.

A total of 4025 seeds (35 for each one of the 109 coastal and 6 hybrid families) were selected from parents throughout the species’ natural distribution in western Oregon and Washington, ensuring a wide variation of temperature and moisture in the sample collection (Fig. 1). In December 2018, seeds were cold stratified for 1 month at 4 °C before planting and later sown in SC10 containers (8.25 × 1.5″, Ray Leach Cone-tainers-SC10 Super, Stuewe & Sons, Inc., Tangent, OR, USA) containing a soil mixture made up of 1-part sphagnum peat moss, 1-part coarse vermiculite and 1-part horticultural perlite. Seedling containers were placed in racks (30.5 cm × 37.6 cm each), and groups of racks were on adjacent benches (4.5 m^2^ each) in a completely randomized design in the same greenhouse room at the Northern Arizona University (NAU) greenhouse facility (Flagstaff, Arizona). Seeds were watered three times a week until germination. After germination, seedlings were watered daily until secondary needles appeared, for about 4 weeks. Seedlings were fertilized once weekly during the months of April through July of 2019, using a balanced water-soluble fertilizer at a concentration of 60 ppm. The greenhouse temperatures were maintained between 15 and 23 °C during growing season, from April to September. To follow the natural process of dormancy in the species, we mimicked dormancy by placing trees in a cool greenhouse from October (2019) to March (2020). During this period, the greenhouse temperatures varied between 7 and 15 °C. All seedlings were then transplanted into D-40 pots (0.65 L) in the summer of 2020.

### Survival and growth measurements

Germination and seedling survival were evaluated in March 2020. Five coastal families with less than 5 % germination rate were excluded for posterior experiments (all other families had an average germination rate of 80 % with an average of 22 individuals per family). Heights for all remaining seedlings (2474 total) growing in the greenhouse were measured in October 2020 when seedlings were 21 months old. The height of the uppermost bud, measured from the stem base, was recorded for each seedling. All measurements were taken in millimeters using a standard meter stick.

### Heat stress experiment

Needles were subjected to a range of treatment temperatures to simulate heat stress, upon which conductivity of electrolyte leakage was measured in microsiemens and compared with the same measurement after 100 % damage (Ruter 1993; Agarie *et al.* 1995). This approach is similar to the use of electrolyte leakage to study freeze tolerance (Sutinen *et al.* 1992; Lu *et al.* 2007), but in our study heat is used instead of below-freezing temperatures. A pilot study was conducted to develop the protocol and assess the viability of the experiment. In this pilot study, 2 individuals from each of three different families were subjected to eight different treatment temperatures in increments of 5 °C (30–65 °C; data not shown). Upon review, the 30 °C treatment was excluded from subsequent experiments due its similarity with the 35 °C treatment.

The morning of each treatment, four needles were picked from one individual of each of the 100 selected families. Needles were placed in separate white paper envelopes and labeled accordingly. Each of the four needles per individual was then cut into 1 cm segments, with both ends having been cut, and placed into a corresponding labelled test tube. Next, 1 mL of laboratory-grade deionized water was added to each test tube and capped with a square of aluminium foil to prevent dehydration during treatments. Each treatment group of 100 individuals was then submerged into a Precision GP10 Thermo Scientific water bath (Thermo Fisher Scientific, Waltham, MA, USA) at the desired treatment temperature (35, 40, 45, 50, 55, 60, 65 °C) for 60 min each. After removing the treatment group(s) from the bath, 4 mL of additional deionized water was added to each test tube and the aluminium foil caps were replaced. Each treatment group was then placed on a KJ-201BD Orbital Shaker at 150 RPM for 40 min. Following the shaking, each treatment group was placed in a room temperature (25 °C) bath for 30 min. Using a Fisherbrand conductivity meter (Thermo Fisher Scientific), conductivity of the solution in each test tube was measured and recorded. To determine the total number of electrolytes for each sample, each treatment group was placed in a Fisher Scientific SterilElite24 autoclave at 121 °C, for the full 50-min cycle. After one final manual inversion of each tube, final conductivity of the solution in each test tube was then measured and recorded to represent 100 % leaf damage. The following formula was used to calculate the percentage of electrolyte leakage for each individual temperature treatment after taking the conductivity after 100 % damage into account:


$$\begin{array}{l} \%\;electrolytic\;leakage=(conductivity\;at\;selected\;Temp\\ /conductivity\;at\;121{}^{\circ }C)\times 100 \end{array}$$

This process was repeated for five individuals from each of the 100 families, in all seven treatment temperatures. Percent electrolyte leakage was then averaged among the five individuals from each family.

### Carbon isotope and leaf nitrogen analysis

Carbon isotope discrimination (∆^13^C) and nitrogen concentrations (%N) were measured in newly developed needles in 22-month-old seedlings from 105 families, with a total of 525 individual seedlings (same families used for the heat stress experiment plus five additional ones). These traits were measured using elemental analyzer-continuous flow isotope ratio mass spectrometry with an Erba NC 2100 EA (Carlo) interfaced to a Finnigan Delta Plus XL IRMS (Thermoquest, San Jose, CA.) at the Colorado Plateau Stable Isotope Laboratory located at NAU.

At least six needles were collected from five individuals per each of the 105 families. Needles were placed into labeled paper coin envelopes and placed in a SHEL LAB Forced Air Oven at 65 °C for 48 h to dry. Needles were then placed in 2 mL tubes with one grinding ball (4 mm) bearing and placed in a SPEX SamplePrep 1600 MiniG mill for 2 min. Following an additional 24-h drying period, ground needles were then rolled into tin capsules (4 × 6 mm) with a target sample weight of 2.00 mg ± 0.100 mg. Upon receiving the data from the carbon isotope analysis, δ^13^C data were then used to calculate carbon isotope discrimination (∆^13^C) using the following equation (Farquhar *et al.* 1989):


$$\begin{array}{l} \Delta^{13}C\;\;\;=\frac{-0.008+\left[\frac{\delta^{13}C}{-1000}\right]}{1-\left[\frac{\delta^{13}C}{-1000}\right]}\times 1000 \end{array}$$

Here, δ^13^C is the ratio of the stable carbon isotope in Douglas-fir needle tissues, and −0.008 is the approximate δ^13^C of atmospheric CO_2_ established in the Pee Dee Belemnite reference standard.

### Statistical analyses of phenotypic traits

A one-way analysis of variance (ANOVA) was used to test for significant differences among families for growth, heat tolerance for each of the seven temperature treatments (35, 40, 45, 50, 55, 60, 65 °C), carbon isotope discrimination and leaf nitrogen. The analysis was repeated three times, including only coastal, only hybrids, and coastal and hybrid families. We used mixed-model ANOVA to test for differences in measured traits among the two varieties with variety as a fixed effect and family as a random effect using the lme4 package. The residuals of the models approximated a normal distribution. A Tukey’s Honestly Significant Difference (Tukey’s HSD) post-hoc test was used for pairwise comparisons of all families. Relationships among family trait means were evaluated using correlation and regression analyses. All data analyses were carried out using R studio version 1.1.442 (packages Hmisc and ggcorrplot) and JMP PRO 15.2.

### Correlations among geographic, environmental variables and phenotypic traits

Seed source elevation data were obtained using coordinates and a digital elevation model (DEM) in GIS. Climate data were obtained using ClimateNA software that downscales PRISM monthly climate data from 1962 to 1990 to scale-free point data, allowing for a more accurate representation of maternal tree climate variables (Wang *et al.* 2016). From the available data, 23 annual climate variables and 3 geographic variables were obtained and included in the final dataset alongside the phenotypic traits that were measured in the greenhouse and the lab ([see Supporting Information—Tables S1 and S2]). Correlations among geographic, environmental and phenotypic variables were analyzed in R (version 4.0.4).

### DNA extraction and genotyping

Douglas-fir seeds were soaked in a 7:3 solution of water and 3 % hydrogen peroxide for 12 hours. Megagametophyte haploid tissues were dissected from ten half-sib individuals for each family and pooled together to infer the maternal genotype. DNA was extracted using the Qiagen DNeasy mini-prep Plant kit and an Eppendorf automated pipetting workstation, with a lab protocol that included one day of tissue lysis and incubation at 96°C, followed by several steps of precipitation and filtering. The quality and concentration of DNA were assessed using a NanoDrop Spectrophotometer and a Qubit 2.0 Fluorometer. Genotyping for all samples was done with 20,397 single-nucleotide polymorphism (SNPs) markers obtained from a custom-designed gene-based Illumina Infinium SNP array (Weiss *et al.* 2020; De La Torre *et al.* 2021). A total of 14,980 SNPs was retained for posterior analyses. This array was developed to represent genome-wide variation by containing both coding and non-coding regions of the genome. To assure species-wide levels of genetic diversity, whole-genome resequencing data from individuals across the species’ geographic range were used as input for SNP array construction (Weiss *et al.* 2020). GenomeStudio Genotyping Module v.2.0 (Ilumina 2016) was used to call genotypes, filter and generate genotyping statistics for all samples and SNPs. SNPs with a call frequency ≤0.65 and a call rate ≤0.8 were not included. Further, markers were filtered out based on minor allele frequency (>0.01) to remove all monomorphic and low-quality SNPs. SNP functional annotations were obtained from aligning against the full NCBI non-redundant protein sequences database (nr) using BLASTP (e-value < e−^10^), and by using the Douglas-fir’s version Psme.1.0 genome annotations (https://treegenesdb.org/FTP/Genomes/Psme/v1.0/annotation).

### Population structure

Population structure in the dataset was evaluated using a Principal Component Analysis (PCA) in TASSEL v.5 (Bradbury *et al.* 2007). In addition, the Python 2.X FastSTRUCTURE (Raj *et al.* 2014) algorithm was used for posterior inference of the number of clusters (*K*) that better explain the population genetic structure of the dataset. Models in fastSTRUCTURE were replicated 10 times for each *K* value from 1 to 10 with default prior parameters and random seed numbers for each run. All runs were combined using CLUMPP (Jakobsson *et al.* 2007) and ancestry barplots were generated in R.

### Univariate and multivariate genome-wide association studies

General and mixed linear models (GLM and MLM) were implemented in the univariate GWAS analyses in TASSEL v.5 (Bradbury *et al.* 2007). The first two principal components of a PCA were used as co-variates to control for population structure in the GLM model, and a kinship matrix was used to account for relatedness in the MLM model (Bradbury *et al.* 2007). Associations between SNP markers and heat, water use efficiency and growth-related traits were tested using univariate linear mixed models (uLMM) and multivariate linear mixed models (mvLMM) in GEMMA v0.98.3 (Zhou *et al.* 2013). In contrast to the uLMM method, mvLMM associates multiple phenotypic traits with all markers simultaneously, while controlling for population structure and relatedness. To run GEMMA, PLINK binary ped format was generated using PLINK v.1.9 software for association analysis. Bonferroni and false discovery rate (FDR) (<0.05) were applied for correction for multiple testing to identify significant SNPs.

### Functional gene annotations

Genomic positions of significant SNPs were investigated to identify the annotated genes by scanning the genomic VCF files of Douglas-fir. Subsequently, the identified significant SNPs were annotated using annotation files downloaded from TreeGenes (https://treegenesdb.org/TripalContactProfile/588450). The annotation was confirmed using some other approaches such as pfam (Finn *et al.* 2014), blastp (Johnson *et al.* 2008) and BlastKOALA (Kanehisa *et al.* 2016b). Pfam was run using HMMER (Finn *et al.* 2011) at default parameters with e-value 1.0 to search proteins families. The blastp was run at expected threshold-0.05; matrix-BLOSUM 62; database- non-redundant protein sequence (nr) to search the similar hits. The BlastKOALA at KEGG (Kanehisa *et al.* 2016a) was performed for protein pathways and annotations. Identical matching genes were chosen to identify annotations and KEGG pathways.

## Results

### Growth measurements

A total of 2474 seedlings were evaluated for height, and significant differences were found among families and varieties. Family-averaged height (mm) varied from 61.3 (family 8049) to 188.75 (family 3027). Height measurements significantly differed among the coastal (*F* = 14.5, *P*-value = <0.0001) and hybrid Douglas-fir families (*F* = 25.73, *P*-value = <0.0001; Table 1); with coastal families growing faster and taller (128.11 ± 29.17) than families from the hybrid variety (86.41 ± 27.93; *F* = 30.211, *P*-value < 0.0001; Table 1). No significant correlations were found between seedling height and geographic variables (latitude, longitude) over all seedlings.

**Table 1.** Differences in height, heat tolerance (electrolyte leakage from 35 to 65 °C), carbon isotope discrimination (∆^13^C), and foliar nitrogen concentration (%N) and C:N ratio among coastal families, among hybrid families, and between coastal and hybrid varieties based on ANOVA results. Electrolytic leakage was measured at 35, 40, 45, 50, 55, 60 and 65 °C .

	Coastal families		Hybrid families		Coastal and hybrids	
Trait	*F* value	*P* value	*F* value	*P* value	*F* value	*P* value
Height	14.50	<0.0001	25.73	<0.0001	30.211	<0.0001
T35C	1.511	0.003	1.218	0.335	0.601	0.439
T40C	1.728	0.000	3.497	0.040	0.479	0.490
T45C	1.588	0.001	2.605	0.087	1.358	0.246
T50C	1.396	0.015	2.161	0.132	3.362	0.069
T55C	0.907	0.713	0.793	0.515	6.150	0.013
T60C	1.210	0.108	0.321	0.809	1.442	0.232
T65C	1.241	0.081	0.930	0.449	0.134	0.714
∆^13^C	2.691	<0.0001	11.356	<0.0001	5.079	0.026
%N	4.530	<0.0001	5.517	0.018	8.695	0.003
C:N	5.161	<0.0001	6.873	0.0005	15.589	<0.0001

### Heat stress experiment

**Figure 2.**
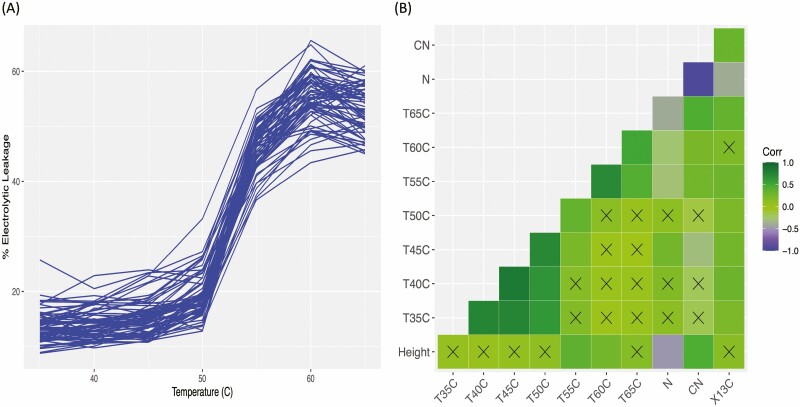
Results of heat stress experiments and correlations among carbon isotope discrimination (an index of water use efficiency) and heat traits measured in this study. (A) Average foliar % electrolytic leakage of 100 Douglas-fir families (blue lines) at seven temperature exposures; (B) Heatmap showing correlations among height, heat tolerance at seven temperatures (T35C-T65C), %N (N), C:N ratio (CN), carbon isotope discrimination (X13C). Colour legend indicates the Pearson’s correlation value (*r*). Crosses indicate non-significant correlations.

**Figure 3.**
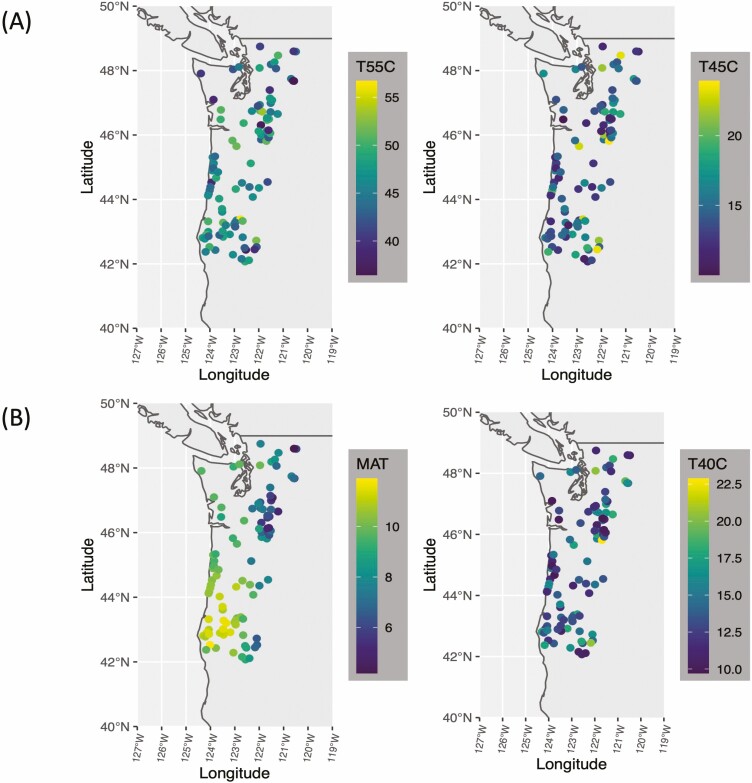
Variation in heat tolerance (measured through % electrolytic leakage) across geographical origin of coastal and hybrid Douglas-fir families in Oregon and Washington. (A) Geographic variation in % electrolytic leakage at 55 °C (T55C) and 45 °C (T45C); (B) Geographic variation in mean annual temperature (MAT) and % electrolytic leakage at 40°C (T40C).

**Figure 4.**
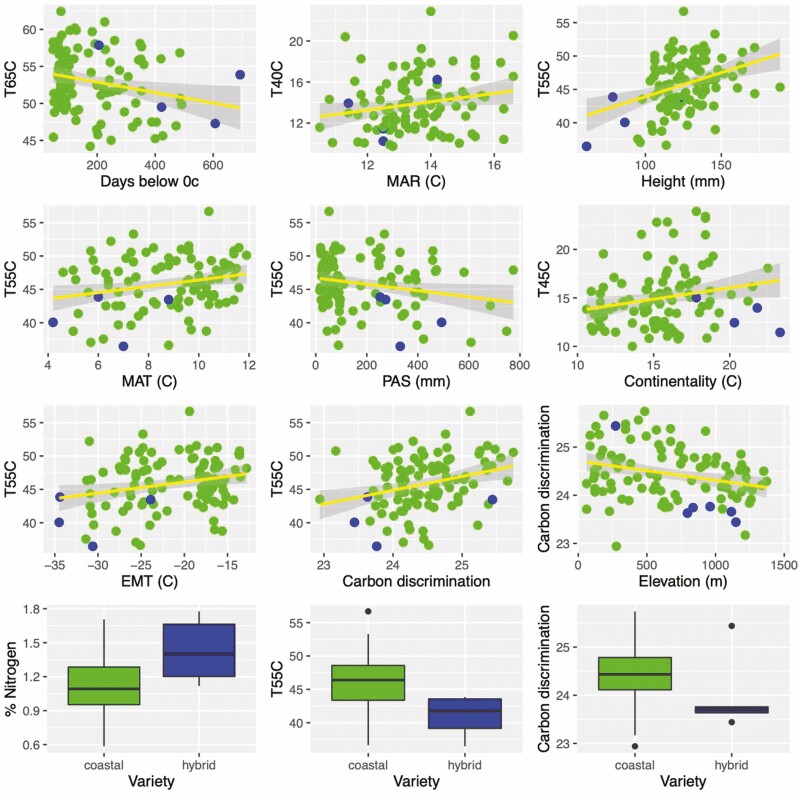
Correlations among heat tolerance, carbon isotope discrimination (an index of water use efficiency), growth and environmental variables in Douglas-fir families. Coastal families are shown in green and intervarietal hybrids in blue. Heat tolerance was measured by % electrolytic leakage at seven temperatures (T35C–T65C). Bottom boxplots show differences between coastal and hybrid families in % nitrogen, electrolytic leakage at 55 °C (T55C) and carbon isotope discrimination. A higher percentage of electrolytic leakage indicates a lower tolerance to heat. All correlations are significant with a *P*-value < 0.001. Environmental variables included mean annual temperature (MAT), mean annual radiation (MAR), precipitation as snow (PAS), extreme minimum temperature (EMT), continentality (temperature difference between mean warmest and mean coldest month) and degree days below 0 °C.

The evaluation of Douglas-fir families suggested significant genetic variation in heat tolerance at different temperatures across geographical origin of coastal and hybrid Douglas-fir families (Figs. 2 and 3; Table 1). Significant differences in electrolyte leakage were found among coastal families at temperatures 35, 40, 45 and 50 °C (*P*-value < 0.05, Table 1). In hybrid families, differences in electrolyte leakage were only significant at 40°C (F = 3.497, p-value= 0.0401, Table 1). Across all families, extensive damage of Douglas-fir foliage occurred between 50°C and 60°C (Fig. 2). Hybrid families had significantly lower electrolyte leakage than coastal families at 55°C (p-value <0.05; Table 1; Fig. 4).

All correlations among environmental variables and between environmental variables and traits can be found in Fig. 4 and [see Supporting Information—Figs. S1 and S2]. On average, families from higher elevation, more inland locations with drier and colder weathers had less heat induced electrolyte leakage than families from lower elevation, coastal locations with warmer and more humid climates.

### Carbon isotope and leaf nitrogen analyses

Significant differences in ∆^13^C (*F* = 2.691, *P*-value < 0.0001), %N (*F* = 4.53, *P*-value < 0.0001) and C:N (*F* = 5.161, *P*-value = <0.0001) were found among coastal Douglas-fir families (Table 1). Hybrid families also had significant differences in all above traits (*P*-value < 0.05, Table 1). Family-averaged ∆^13^C varied from 22.94 ‰ (family 6089) to 25.74 ‰ (family 5097) (mean= 24.47 ± 0.84) among coastal Douglas-fir families. ∆^13^C was positively correlated with electrolytic leakage across all temperatures at which extensive cell damage occurs (Fig. 2). In addition, elevation was negatively correlated with ∆^13^C and C:N (*P*-values of 0.0037, 0.0032, respectively) and positively correlated with %N (*r* = 0.3, *P*-values = 0.002). ∆^13^C, %N and C:N ratio were significantly different between coastal and hybrid families (*P*-values < 0.05; Table 1).

Across all families, height was positively correlated with electrolyte leakage at 55 °C with an *r* of 0.36 and a *P*-value of 0.000197 (Fig. 2), and with electrolyte leakage at 60 °C (*r* = 0.25, *P*-value = 0.0133; Fig. 2). In addition, seedling height was negatively correlated with %N and positively correlated with C:N (Fig. 2).

### Genome-wide association studies

A principal component analysis of Douglas-fir families suggested a low population structure in the dataset with the coastal individuals divided between one main cluster and one smaller cluster (Oregon’s southern distribution); and the hybrid individuals located in eastside Washington’s Cascades ([see Supporting Information—Fig. S3]). The fastSTRUCTURE ancestry analysis suggested the mother trees of these hybrid families have mixed ancestry from the coastal and interior varieties ([see Supporting Information—Fig. S4]). Univariate and multivariate GWAS analyses were repeated twice, the first time for all dataset (coastal and hybrids), and a second time only for coastal families. Population structure in the datasets was accounted for in the GWAS analyses to avoid the presence of false positives. Univariate and multivariate GWAS investigated the presence of significant associations among 11 heat, drought, and growth-related traits and 14,980 genetic markers. Results of the univariate and multivariate GWAS of coastal and hybrids identified 35 significant associations between 6 traits (electrolytic leakage at 35, 40 and 45 and 50 °C; C:N ratio and height) and 31 genes (Table 2 and [see Supporting Information—Table S3]). Most genes were associated with only one trait, except for transcription factor VOZ1 PSME_15499 gene, which was associated with electrolytic leakage at 35 and 50 °C; and uncharacterized gene PSME_42829, which was associated with electrolytic leakage at 40 and 45 °C. The proportion of phenotypic variance explained by each significant SNP marker (based on the GLM model) varied from 22 to 26.4 % ([see Supporting Information—Table S3]).

**Table 2.** Functional annotation of the genes of significant SNPs identified by univariate linear mixed models (ULMM), multivariate linear mixed models (MVLMM) at GEMMA, and general linear model (GLM) in TASSEL for coastal and hybrid Douglas-fir. The ‘Trait’ column identifies the heat tolerance at different temperatures, carbon isotope discrimination, nitrogen content, or growth trait; ‘Gene’ column identifies the gene associated with the trait; ‘Similar search ID’ column indicates the genbank or uniport ID identifying similar BLAST hits; ‘SNP’ column indicates the number of associated SNPs; *P*-value is the significance level; and Annotation and KEGG provide the functional annotation for candidate genes. Genes in bold were identified as associated with growth, cold hardiness, phenology and environmental variables in a previous Douglas-fir study (De La Torre *et al.* 2021).

Trait	Gene	Similar Search ID	SNPs	P-value	Annotation	KEGG Pathway/KEGG-Brite/Process
T35C	PSME_20450^††^	KAF7835953.1	1	6.26^−07^	Ecotropic viral integration site protein	—
T35C	PSME_48755^††^	XP_031386554.1	1	3.16^−06^	F-box/FBD/LRR-repeat protein	—
T35C	PSME_04475^††^	XP_024384147.1	1	8.06^−07^	F-box/Kelch-repeat protein	—
T35C	PSME_39629^††^	XP_031480439.1	1	1.36^−06^	Inositol-tetrakisphosphate 1-kinase 3-like isoform	Inositol phosphate metabolism, phosphatidylinositol signalling system pathways
T35C	psme_t025434|m.78800^††^	—	1	1.86^−06^	—	—
T35C	PSME_15499^††^	XP_010260370.1	1	2.89^−06^	Transcription factor VOZ1	Abiotic and biotic stress
T40C	PSME_42829^††^	CAN68428.1	1	3.21^−06^	Uncharacterized protein	—
T45C	PSME_42829^††^	CAN68428.1	1	2.11^−06^	Uncharacterized protein	—
T50C	PSME_15499 ^†^	XP_010260370.1	1	2.69^−06^	Transcription factor VOZ1	Abiotic and biotic stress
CN	PSME_31599^††^	XP_031503996.1	1	1.19^−06^	Reticulon-like protein B1	Membrane trafficking
Height	**PSME_27219** ^†^	XP_039125026.1	1	1.14^−08^	Cyclin-dependent kinase C-2 isoform	Cell cycle control
Height	PSME_27198 ^†^	XP_024396643.1	1	2.40^−08^	Nucleolin-like	Ribosomal biogenesis
Height	**PSME_47504** ^†^	ADB97926.1	1	1.45^−07^	Thaumatin-like protein L2	—s
Height	PSME_29636 ^†^	XP_023884839.1	1	1.74^−07^	Uncharacterized protein	—
Height	PSME_48306^†^	-	1	1.74^−07^	—	—
Height	**PSME_35875** ^†^	XP_030930392.1	1	1.79^−07^	NAD(P)H dehydrogenase	Ubiquinone and other terpenoid-quinone biosynthesis pathway
Height	**PSME_47942** ^†^	XP_038710372.1	1	2.35^−07^	Cysteine-rich repeat secretory protein	Salt stress
Height	**PSME_47800** ^†^	XP_014505538.1	1	2.56^−07^	Inactive purple acid phosphatase	Growth and development
Height	PSME_52709 ^†^	—	2	2.78^−07^	—	—
Height	PSME_42675 ^†^	ABR16422.1	1	3.71^−07^	Unknown	—
Height	PSME_45982 ^†^	XP_024973942.1	1	4.38^−07^	UPF0481 protein At3g47200-like	—
Height	**PSME_01087** ^†^	XP_017217789.1	1	5.03^−07^	AT-hook motif nuclear-localized protein	DNA replication and repair
Height	PSME_02019 ^†^	XP_009346468.1	1	6.00^−07^	Uridine-cytidine kinase C-like	Pyrimidine metabolism, metabolic pathways
Height	PSME_33611 ^†^	XP_010920821.1	1	8.23^−07^	Protein PMR5	Biotic stress
Height	**PSME_00292** ^†^	NP_013268.1	1	1.00^−06^	Ubiquitin-ribosomal 40S subunit protein	Ubiquitin mediated proteolysis
Height	**PSME_28922** ^†^	QBI90547.1	1	1.29^−06^	Flavanone-3-hydroxylase	Flavonoid biosynthesis, biosynthesis of secondary metabolites pathways
Height	PSME_01405 ^†^	XP_038985456.1	1	1.40^−06^	Putative disease resistance protein	Plant–pathogen interaction
Height	**PSME_32617** ^†^	XP_011077233.1	1	1.48^−06^	Phosphoenolpyruvate carboxylase kinase 2	Calcium signaling pathway, biotic stress
Height	PSME_42381 ^†^	XP_021276483.1	4	2.11^−06^	Uncharacterized protein	—
Height	**PSME_04717** ^†^	ABF58895.1	1	2.46^−06^	GABA aminotransferase	Alanine, aspartate and glutamate metabolism pathway
All	PSME_03650 ^†††^	NP_012017.1	1	2.31^−06^	Mitochondrial 54S ribosomal protein	—
All	PSME_42829 ^†††^	CAN68428.1	1	7.25^−06^	Uncharacterized protein	—
All	PSME_15499 ^†††^	XP_010260370.1	1	2.75^−06^	Transcription factor VOZ1	Abiotic and biotic stress
All	PSME_15952 ^†††^	XP_024168655.1	1	2.87^−06^	mRNA turnover protein 4	Ribosome biogenesis pathway

^†^Univariate linear mixed models (ULMM) at GEMMA.

^††^General linear model (GLM) analysis at TASSEL.

^†††^Multivariate linear mixed models (MVMM) at GEMMA, All = phenotypes including height, 35, 40, 45, 50, 55, 60, 65 °C, N, C: N, ∆^13^C.

The GLM analyses of coastal families identified 12 significant SNPs (*P*-value < 3.00E^−06^), 10 of them were associated with electrolytic leakage at 35 °C, and 2 with C:N ratio. Those significant SNPs matched seven genes in seven different scaffolds in the genome of Douglas-fir ([see Supporting Information—Table S4]; Table 3). The proportion of phenotypic variance explained by each significant SNP marker varied from 21.7 to 27.3 % ([see Supporting Information—Table S4]). Subsequently, univariate linear mixed model (uLMM) and multivariate linear mixed model (mvLMM) approaches were performed in GEMMA to identify significant SNPs. MvLMM identified 10 significant SNPs (*P*-value <3.00E^−06^) ([see Supporting Information—Table S5]); and uLMM identified three SNPs (*P*-value < 3.00E^−06^) associated with electrolytic leakage at 35°C, 40°C, and 45°C ([see Supporting Information—Table S6]). The GWAS results from both datasets (coastal plus hybrids, and only coastal) identified seven common genes, identified as F-box proteins, reticulon-like protein, inactive purple acid phosphatase and a gene involved in the inositol phosphate metabolic pathway (Tables 2 and 3). Other genes were involved in the biosynthesis of primary and secondary metabolites, Ubiquitin-mediated proteolysis, transport, transcription and ribosome biogenesis (Tables 2 and 3). Ten of the genes associated with height in this study (Table 2) were found to be associated with growth, cold hardiness, phenology and environmental variables (continentality and/or days below zero degrees) in a previous study in Douglas-fir (De La Torre *et al.* 2021). Seeds from some of the mother trees in the 2021 study were grown and analyzed in the present study.

**Table 3.** Functional annotation of the genes of significant SNPs identified by univariate linear mixed models (ULMM), multivariate linear mixed models (MVLMM) at GEMMA, and general linear model (GLM) in TASSEL for coastal Douglas-fir (*Pseudotsuga menziesii*). The ‘Trait’ column identifies the associated heat tolerance at different temperatures, carbon isotope discrimination, nitrogen content, or growth trait; ‘Gene’ column identifies the gene associated with the trait; ‘Similar search ID’ column indicates the genbank or uniport ID identifying similar BLAST hits; ‘SNP’ column indicates the number of associated SNPs; *P*-value is the significance level and ‘Annotation’ provides the functional annotation for candidate genes.

Trait	Gene	Similar search (ID)	SNP	*P*-value	Annotation
T35C	PSME_39629^††^	XP_031480439.1	1	1.73^−06^	Inositol-tetrakisphosphate 1-kinase 3-like isoform
T35C	PSME_20450^††^	KAF7835953.1	1	3.51^−07^	Ecotropic viral integration site protein
T35C	PSME_48755^††^	XP_031386554.1	2	9.79^−-07^	F-box/FBD/LRR-repeat protein
T35C	PSME_04475^††^	XP_024384147.1	1	1.13^−06^	F-box/Kelch-repeat protein
T35C	PSME_00998^††^^*^	XP_031497232.1	1	1.73^−06^	Pentatricopeptide repeat-containing protein
T35C	PSME_47800^††^	XP_014505538.1	1	1.73^−06^	Probable inactive purple acid phosphatase
T40C	PSME_42829^†^	CAN68428.1	1	1.97^−07^	Uncharacterized protein
T45C	PSME_42829^†^	CAN68428.1	1	1.14^−07^	Uncharacterized protein
CN	PSME_31599^††^	XP_031503996.1	1	1.92^−06^	Reticulon-like protein B1
All	PSME_03650 ^†††^	NP_012017.1	1	1.74^−07^	Mitochondrial 54S ribosomal protein
All	PSME_36695 ^†††^^*^	XP_024401260.1	1	5.39^−07^	Protein EXECUTER2, chloroplastic-like isoform
All	PSME_42829^†††^	CAN68428.1	1	1.25^−07^	Uncharacterized protein
All	PSME_15952^†††^	XP_024168655.1	1	2.68^−07^	mRNA turnover protein

^†^Univariate linear mixed models (ULMM) at GEMMA.

^††^General linear model (GLM) analysis at TASSEL.

^†††^Multivariate linear mixed models (MVMM) at GEMMA; All = phenotypes including height, 35, 40, 45, 50, 55, 60, 65 °C, N, C: N, ∆^13^C; *Not found as candidate genes in Table 2.

## Discussion

### Differences in heat tolerance among Douglas-fir families and varieties

Our heat stress experiment included foliar temperatures (50–55 °C) similar to those reported in forests of Douglas-fir in the Pacific Northwest region during the unprecedented ‘heat dome’ event in summer of 2021 (Philip *et al.* 2021). Thus, our results are relevant to emerging extreme heat events that are expected to increase in the future with climate warming. Exposure to high temperatures caused substantial tissue damage across all families. We found differences among families at moderate exposure (40 and 45 °C) but not at temperatures above 55 °C, suggesting that exposure to such extreme temperatures causes irreversible damage across all families due to denaturing of proteins. At such high temperatures, seedling survival might depend on maintaining high transpiration rates for heat dissipation (Kolb and Robberecht 1996). However, this might be challenging under more frequent and intense droughts predicted with climate change. Differences in electrolyte leakage at moderate temperatures among Douglas-fir families were attributed to an adaptation to source climate in an earlier study (Marias *et al.* 2016). Lower electrolyte leakage in hybrids at 55 °C compared with the coastal variety suggest a higher tolerance to these temperatures for hybrids and should be explored further to test if results of electrolyte leakage translate to enhanced performance under field conditions. In addition, future studies should test electrolyte leakage at different temperatures under high and low water conditions to determine tree response to an interaction of heat and water stress.

### Higher elevation Douglas-fir families have higher water use efficiency

Carbon isotope discrimination significantly varied among coastal families, among hybrid families and between coastal and hybrid varieties. This result is consistent with earlier studies that found differences among Douglas-fir varieties in carbon isotope discrimination (Zhang *et al.* 1993; Anekonda *et al.* 2004). Our results suggest an increase in water use efficiency and photosynthetic capacity with an increase in seed source elevation. Lower carbon isotope discrimination (higher water use efficiency) in families from higher elevations could be a result of higher photosynthetic capacity, as suggested by a significant positive correlation between source elevation and %N. Our results of a decrease in carbon isotope discrimination and an increase in foliar nitrogen with elevation are consistent with an earlier study on riparian tree species in southern Utah (Sparks and Ehleringer 1997). Future studies should focus on direct measurements of leaf level gas exchange to gather information about differences in net carbon assimilation and stomatal conductance among Douglas-fir families.

### Correlations between heat stress, water use efficiency and growth

Interestingly, families with higher water use efficiency also had higher tolerance to heat stress. We found a positive correlation between electrolyte leakage after exposure to high temperatures and carbon isotope discrimination (under well-watered conditions), suggesting that families with high tolerance to extreme temperatures were also better able to control water loss by stomatal closure. In addition, faster growing families were more susceptible to heat damage as evidenced by a positive correlation between tree height and electrolyte leakage after exposure to temperatures between 55 and 65 °C (Fig. 2); this suggests a trade-off between growth and heat tolerance. Families with the highest electrolyte leakage under extreme heat stress were from locations with a higher number of frost-free days, less precipitation as snow, fewer days below 0 °C, and warmer average temperatures, both annually and during the coldest and warmest months (Supporting Information—Fig. S2). Our results suggest that slow-growing families from colder climates are more tolerant to heat stress than fast-growing families from warmer climates. Previous research shows trade-offs between tree growth and cold hardiness in Douglas-fir; however, trade-offs between tree growth and heat/drought resistance have not been clear (Darychuk *et al.* 2012). We used tree height to represent overall growth; however, radial growth is also an important metric of growth which is responsive to drought (Sergent *et al.* 2014). Overall, the results of our study indicate that intervarietal hybrid families with slower growth and higher water use efficiency had greater tolerance of extreme heat, which suggests that they might be better adapted to future water conditions than the coastal variety given that high temperatures are an emerging environmental stress and threat to global forests (Marias *et al.* 2016; Philip *et al.* 2021).

Previous studies of Douglas-fir found a trade-off between drought tolerance and growth (St. Clair and Howe 2007) and cold hardiness and growth (De La Torre *et al.* 2021). Our results suggest that fast-growing families are less tolerant to heat stress as well (Fig. 2), so it appears that all three stress-tolerance traits (heat, drought and cold tolerance) are inversely correlated to height in Douglas-fir. In a previous greenhouse study by Bansal *et al* (2016), cold hardiness and drought tolerance were found to be linked in coastal Douglas-fir. Trees from regions with cold winters had higher cold and drought tolerance likely due to the overlapping adaptations and their ability to deal with winter desiccation (Bansal *et al.* 2016). The results of our experiment support this finding. In addition, our results indicate that the colder the seed source environment (whether it be in terms of the number of frost-free days or mean coldest month temperature), the more resistant foliar cells are to extreme heat, which indicates greater heat tolerance ([see Supporting information—Fig. S2]).

### Role of hybridization in heat tolerance and water use efficiency in Douglas-fir

Our results indicate that hybrid families had higher heat tolerance and water use efficiency compared to coastal families (Fig. 4). As inferred by the proportions of interior and coastal ancestries in the mother trees, seedlings in our study are not first-generation hybrids but most likely are the product of several generations of hybridization and introgression among the interior and coastal varieties ([see Supporting Information—Fig. S4]). Strong differences in heat tolerance and water use efficiency were evident in families in which the mother trees had a higher ancestry from the interior variety (all hybrid families except for family 5029 which had a higher coastal ancestry, [see Supporting Information—Fig. S4]). Therefore, we suggest that adaptive introgression from the interior variety might have resulted in natural intervarietal hybrids with higher water use efficiency and tolerance to heat. However, conclusive evidence requires further drought and heat tolerance studies including all species’ varieties (coastal, interior, Mexican) and hybrid classes (F1s, F2s, backcrosses). It is also important to consider that our study was restricted to the Washington Cascades, but natural hybrids are also reported in British Columbia. Therefore, our results might not represent all hybrid individuals naturally growing across the species’ geographic range. Indirect evidence from a common garden study growing the same families in Oregon (St. Clair *et al.* 2005) suggests that these hybrids might also have higher cold hardiness. Examples of adaptive introgression between widely distributed, mostly outcrossing, closely related, forest tree species or varieties have been reported in *Populus tremula* (Rendón-Anaya *et al.* 2021), *Quercus robur* × *Q. petraea* (Leroy *et al.* 2020), *Populus balsamifera* × *P. trichocarpa* (Suarez-Gonzalez *et al.* 2016), *Picea glauca* × *P. engelmannii* (De La Torre *et al.*2014a, 2014b), among others. The results of our study suggest that hybridization might be a source of pre-adapted alleles to warming climatic conditions and should be considered for planting under increasingly arid conditions.

### Functional annotation of candidate genes

Trees respond to abiotic stress in numerous ways, including physiological, genetic, cellular and morphological changes (Kijowska-Oberc *et al.* 2020). In this study, we identified 32 candidate genes associated with water use efficiency, heat tolerance and growth. These genes were involved in many important biological processes such as primary and secondary metabolism (inositol phosphate metabolism, ubiquinone and other terpenoid-quinone biosynthesis, pyrimidine metabolism, alanine, aspartate and glutamate metabolism); abiotic, biotic stress and signalling; DNA replication and repair; transport; among other processes (Tables 2 and 3).

From the genes associated with heat stress, we identified PSME_48755 and PSME_04475, F-box/kelch-repeat candidate genes associated with heat tolerance at 35 °C. F-box proteins play critical roles in plant responses to biotic/abiotic stresses and plant developmental process (Baute *et al.* 2017; Wei *et al.* 2021). F-box gene transcripts in *Arabidopsis* highly accumulated in roots and altered their response under drought stress conditions. In addition, F-box mutant plants displayed better growth under drought stress conditions compared to the wild type with a reduced accumulation of H_2_O_2_ and malondialdehyde (MDA) (Rao and Virupapuram 2021). F-box proteins (FBX92) had been linked to reduced leaf growth, since overexpression of At*FBX92* resulted in smaller leaves than the wild type in *Arabidopsis thaliana* (Baute *et al.* 2017).

Our results also identified an inositol-tetrakisphosphate 1-kinase 3-like candidate gene (PSME_39629) associated with electrolytic leakage at 35 °C. Inositol signalling plays a crucial role in various aspects of plant growth and adaptation (Jia *et al.* 2019). Inositol phosphate kinases (IPK1) play important roles in diverse cellular processes by functioning as structural and functional cofactors, regulators and second messengers (Shears *et al.* 2012). In the study of Zhang *et al.* (2020), transgenic expression of ThIPK2 (an inositol polyphosphate kinase gene) in wheat led to improved drought tolerance. Compared to the wild-type (WT) plants, the authors identified that transgenic plants showed higher seed germination rates, better developed root systems, a higher relative water content (RWC), total soluble sugar content and less cell membrane damage under drought stress conditions (Zhang *et al.* 2020).

Candidate gene PSME_47800, identified as inactive purple acid phosphatase (PAPs) protein was associated with electrolytic leakage at 35 °C and height. In a previous study by our group, we found that this gene was associated with growth, phenology, degree days below 0 °C and continentality in Douglas-fir (De La Torre *et al.* 2021). PAPs proteins play important roles in phosphate (Pi) acquisition, utilization and developmental processes. It has been reported that differential regulation of CaPAPs under different nutrient deficiencies revealed their roles under multiple nutrient stresses, including Pi deficiency. Most of the CaPAPs have prominently expressed in flower and seed development in Chickpea (Bhadouria *et al.* 2017). Finally, transcription factor VOZ1 candidate gene PSME_15499, associated with heat tolerance in our study, was also associated with heat, drought and salt stress in Arabidopsis (Koguchi *et al.* 2017; Song *et al.* 2018).

## Conclusions

Warmer climates bring significant challenges to the survival of Douglas fir populations. While coastal varieties are widely planted due to their fast growth, their low water use efficiency (a trait of mesic-adapted plants) and low tolerance to heat might predispose them to maladaptation to future climate conditions, as suggested by trade-offs between growth, water use efficiency and heat tolerance found in this study. Our study indicates significant genetic variation in water use efficiency (inferred from carbon isotope discrimination), photosynthetic capacity (inferred from %N), growth and tolerance of leaves to heat stress among Douglas-fir families and varieties. High elevation families had higher water-use efficiency and photosynthetic capacity than low elevation families. In addition, families with greater heat tolerance had slower growth and higher water-use efficiency. Intervarietal hybrids, with mixed ancestry from coastal and interior varieties, had higher water use efficiency and higher heat tolerance than coastal families, suggesting hybridization might be a source of pre-adapted alleles to extreme heat events and drought in increasingly warming climate conditions. Due to the long-generation nature of the species, these results should be considered for reforestation and the selection of seeds sources for commercial plantations under arid conditions.

## Supporting Information

The following additional information is available in the online version of this article –

**Table S1.** Key to geographic and environmental traits acquired by ClimateNA (Wang *et al.* 2016).

**Table S2.** Key to physiological traits measured, including tree height and those derived from heat stress and carbon/nitrogen isotope analysis experiments.

**Table S3.** Total numbers of significant SNPs in coastal and hybrid Douglas-fir identified by general linear model (GLM) at TASSEL. The table contains significant SNPs associated with their phenotypic traits, marker ID, genes ID, genomic positions, *P*-value, SNP marker effect size and functional annotation.

**Table S4.** Total numbers of significant SNPs in coastal Douglas-fir identified by general linear model (GLM) at TASSEL. The table contains significant SNPs associated with their phenotypic traits, and similar search IDs, genomic positions, genes, functional annotation and pathways.

**Table S5.** Total numbers of significant SNPs in coastal Douglas-fir identified by multivariate linear mixed model (mvLMM) at GEMMA. The table contains significant SNPs associated with their phenotypic traits, and similar search IDs, genomic positions, genes, functional annotation and pathways.

**Table S6.** Total numbers of significant SNPs in coastal Douglas-fir identified by univariate linear mixed model (uLMM) at GEMMA. The table contains significant SNPs associated with their phenotypic traits, and similar search IDs, genomic positions, genes and functional annotation.

**Figure S1.** Heatmap showing correlations among environmental variables for all 110 Douglas-fir families. Colour legend indicates the Pearson’s correlation value (*r*). Crosses indicate no significant correlation between traits.

**Figure S2.** Heatmap showing correlations among environmental variables and heat tolerance at different temperatures, carbon isotope discrimination, nitrogen content, and growth traits for all Douglas-fir families. Colour legend indicates the Pearson’s correlation value (*r*). Crosses indicate no significant correlation between traits.

**Figure S3.** Principal components analysis (PCA) based on 14,980 SNP markers shows genetic differences between coastal variety (orange) and hybrid (blue) families in the dataset. Each family contains between 5 and 30 individuals.

**Figure S4.** Ancestry estimates suggest the presence of intervarietal hybrids in this study. Mother trees of all families were analyzed as part of larger dataset of 464 individuals including coastal, interior and hybrids with 14,980 SNP markers in fastSTRUCTURE for *K* = 2 (unpublished data). Ancestry barplot showed here only contains the mother trees of individuals included in this study. Intervarietal hybrids show a combination of coastal (orange bars) and interior (blue bars) ancestry, whereas pure coastal individuals show more than 80 % ancestry from the coastal variety (orange bars).

plad008_suppl_Supplementary_Material

## Data Availability

The data available for this study is included in Supporting Information.
