# Supplementary material for: Differences in heat tolerance, water use efficiency and growth among Douglas-fir families and varieties evidenced by GWAS and common garden studies
Source: AoB Plants. 2023 Mar 1;15(2):plad008. doi: 10.1093/aobpla/plad008 (PMC10052383; doi:10.1093/aobpla/plad008)

SUPPORTING INFORMATION
Compton et al. 2023. Differences in heat tolerance, water use efficiency and growth among Douglas-fir families and varieties evidenced by GWAS and common garden studies.

Table S1. Key to physiological traits measured, including tree height and those derived from heat tolerance and carbon/nitrogen isotope analysis experiments.

**Trait ID**	**Trait Name**	**Unit**	**Trait Description**
Height	Tree Height	mm	Tree height collected October 2020
T35C	Electrolyte leakage at 35C	percent	Relative K+ leakage representing cell damage of foliar tissue of at 35C
T40C	Electrolyte leakage at 40C	percent	Relative K+ leakage representing cell damage of foliar tissue of at 40C
T45C	Electrolyte leakage at 45C	percent	Relative K+ leakage representing cell damage of foliar tissue of at 45C
T50C	Electrolyte leakage at 50C	percent	Relative K+ leakage representing cell damage of foliar tissue of at 50C
T55C	Electrolyte leakage at 55C	percent	Relative K+ leakage representing cell damage of foliar tissue of at 55C
T60C	Electrolyte leakage at 60C	percent	Relative K+ leakage representing cell damage of foliar tissue of at 60C
T65C	Electrolyte leakage at 65C	percent	Relative K+ leakage representing cell damage of foliar tissue of at 65C
13C	leaf ^{13}C discrimination (Δ^{13}C)	per mill	
%N	Nitrogen concentration	percent	

Table S2. Key to geographic and environmental traits acquired by ClimateNA (Wang et al 2016).

**Trait ID**	**Trait Name**	**Unit**	**Variable Description**
Lat	Latitude	degrees	From GIS after mapping parents
Long	Longitude	degrees	From GIS after mapping parents
Elev	Elevation	m	From DEM after mapping parents
MAT	Mean annual temperature	degrees C	Mean annual temperature
MWMT	Mean warmest month temperature	degrees C	Mean warmest month temperature
MCMT	Mean coldest month temperature	degrees C	Mean coldest month temperature
TD	Temperature difference between MWMT and MCMT, or continentality	degrees C	Temperature difference between MWMT and MCMT, or continentality
MAP	Mean annual precipitation	mm	Mean annual precipitation
MSP	May to September precipitation	mm	May to September precipitation
AHM	Annual heat-moisture index		(MAT+1)/(MAP/1000)
SHM	Summer heat-moisture index		(MWMT)/(MSP/1000)
DD_0	Degree-days below zero	days	chilling degree days

DD5	Degree-days above 5C	degrees C	growing degree days
NFFD	Number of frost-free days	days	Number of frost-free days
FFP	Frost-free period	days	Frost-free period
bFFP	The day of the year FFP begins	day of year	The day of the year FFP begins
eFFP	The day of the year FFP ends	day of the year	The day of the year FFP ends
PAS	Precipitation as snow	mm	Precipitation as snow
EMT	Extreme minimum temperature	degrees C	Extreme minimum temperature
EXT	Extreme maximum temperature	degrees C	Extreme maximum temperature
Eref	Hargreaves reference evaporation	mm	Hargreaves reference evaporation
CMD	Hargreaves climatic moisture deficit	mm	Hargreaves climatic moisture deficit

Table S3. Total numbers of significant SNPs in coastal and hybrid Douglas-fir identified by general linear model (GLM) at TASSEL. The table contains significant SNPs associated with their phenotypic traits, marker ID, genes ID, genomic positions, P-value, SNP marker effect size and functional annotation.

Trait	Marker	gene/transcript	Scaffold	Position	P_value	marker_Rsq	NCBI_bestHit	Annotation
T35c	seq-rs2420-DF	PSME_20450	JCF7190000004524	267882	6.26E-07	0.263738003	KAF7835953.1	Ecotropic viral integration site protein
T35c	seq-rs4868-DF	psme_t025434|m.78800	JCF7190000009603	263683	1.86E-06	0.246359553	NA	NA
T35c	seq-rs7123-DF	PSME_48755	JCF7190000014050	285741	3.16E-06	0.237771541	XP_031386554.1	F-box/FBD/LRR-repeat protein
T35c	seq-rs7653-DF	PSME_15499	JCF7190000015448	111143	2.89E-06	0.23921908	XP_010260370.1	transcription factor VOZ1
T35c	seq-rs12015-DF	PSME_39629	JCF7190000026436	52784	1.36E-06	0.220210345	XP_031480439.1	Pentatricopeptide repeat-containing protein
T35c	seq-rs15788-DF	PSME_04475	JCF7190000037831	121035	8.06E-07	0.259741091	XP_024384147.1	F-box/FBD/LRR-repeat protein
T40c	seq-rs15169-DF	PSME_42829	JCF7190000035918	631159	3.21E-06	0.235048604	CAN68428.1	uncharacterized protein
T45c	seq-rs15169-DF	PSME_42829	JCF7190000035918	631159	2.11E-06	0.239679842	CAN68428.1	uncharacterized protein
CN	seq-rs185-DF	NA	JCF7190000000559	587504	1.92E-07	0.260652817	NA	NA
CN	seq-rs186-DF	PSME_31599	JCF7190000000559	587976	1.19E-06	0.234110163	XP_031503996.1	reticulon-like protein
CN	seq-rs16341-DF	NA	JCF7190000039386	99102	2.57E-06	0.222635198	Na	NA

Table S4. Total numbers of significant SNPs in coastal Douglas-fir identified by general linear model (GLM) at TASSEL. The table contains significant SNPs associated with their phenotypic traits, and similar search IDs, genomic positions, genes, functional annotation, and pathways.

Trait	Marker	Scaffold	Pos	P-value	marker_Rsq	Gene	Similar Hits	%	Functional Annotation
35°C	seq-rs2420-DF	JCF7190000004524	267882	3.51E-07	0.2737	PSME_20450	KAF7835953.1	67	Ecotropic viral integration site protein
35°C	seq-rs4868-DF	JCF7190000009603	263683	5.47E-07	0.26682	.	.	.	.
35°C	seq-rs7123-DF	JCF7190000014050	285741	9.79E-07	0.25768	PSME_48755	XP_031386554.1	27	F-box/FBD/LRR-repeat protein
35°C	seq-rs15788-DF	JCF7190000037831	121035	1.13E-06	0.25536	PSME_04475	XP_024384147.1	33	F-box/kelch-repeat protein
35°C	seq-rs7124-DF	JCF7190000014050	285762	1.66E-06	0.24932	PSME_48755	XP_031386554.1	27	F-box/FBD/LRR-repeat protein
35°C	seq-rs7017-DF	JCF7190000013755	477988	1.73E-06	0.21764	PSME_00998	XP_031497232.1	51	pentatricopeptide repeat-containing protein
35°C	seq-rs11462-DF	JCF7190000024943	2264564	1.73E-06	0.21764	.	.	.	.
35°C	seq-rs12015-DF	JCF7190000026436	52784	1.73E-06	0.21764	PSME_39629	XP_031480439.1	65	inositol-tetrakisphosphate 1-kinase 3-like isoform
35°C	seq-rs13589-DF	JCF7190000030999	229036	1.73E-06	0.21764	PSME_47800	XP_014505538.1	68	probable inactive purple acid phosphatase
35°C	seq-rs32118-SP	SCAFFOLD37383	117618	1.73E-06	0.21764	.	.	.	.
CN	seq-rs185-DF	JCF7190000000559	587504	6.20E-07	0.27084	.	.	.	.
CN	seq-rs186-DF	JCF7190000000559	587976	1.92E-06	0.25255	PSME_31599	XP_031503996.1	57	reticulon-like protein B1

Table S5. Total numbers of significant SNPs in coastal Douglas-fir identified by multivariate linear mixed model (mvLMM) at GEMMA. The table contains significant SNPs associated with their phenotypic traits, and similar search IDs, genomic positions, genes, functional annotation, and pathways.

Scaffold	Rs	Pos	Allele1	Allele0	Af	P-value	Genes	Similar Search	%	Annotation
JCF7190000001012	seq-rs463-DF	352744	C	G	0.011	4.64E-15	.	.	.	.
JCF7190000019673	seq-rs9456-DF	21863	A	G	0.016	4.42E-10	.	.	.	.
JCF7190000023663	seq-rs10859-DF	137688	A	G	0.011	1.45E-07	.	.	.	.
JCF7190000036028	seq-rs15198-DF	44027	C	G	0.059	1.74E-07	PSME_03650	NP_012017.1	100	Mitochondrial 54S ribosomal protein
SCAFFOLD77442	seq-rs62964-SP	377132	G	A	0.011	2.56E-07	.	.	.	.
JCF7190000011843	seq-rs6049-DF	301310	A	G	0.032	4.93E-07	.	.	.	.
JCF7190000021917	seq-rs10291-DF	119401	A	G	0.452	5.39E-07	PSME_36695	XP_024401260.1	77	Protein EXECUTER2, chloroplastic-like isoform X6
JCF7190000035918	seq-rs15169-DF	631159	G	A	0.091	1.25E-06	PSME_42829	.	.	.
JCF7190000004021	seq-rs2188-DF	157712	C	G	0.038	1.85E-06	.	.	.	.
JCF7190000016262	seq-rs8011-DF	971922	G	A	0.022	2.68E-06	PSME_15952	XP_024168655.1	72.69	mRNA turnover protein

Table S6. Total numbers of significant SNPs in coastal Douglas-fir identified by univariate linear mixed model (uLMM) at GEMMA. The table contains significant SNPs associated with their phenotypic traits, and similar search IDs, genomic positions, genes, and functional annotation.

Phenotype	Scaffold	Rs	Pos	Allele1	Allele0	Af	Beta	P-value	Gene	KEGG Annotation
35°C	JCF7190000013571	seq-rs6971-DF	568899	G	A	0.011	8.81	0.000002	.	.
40°C	JCF7190000035918	seq-rs15169-DF	631159	G	A	0.09	3.34	1.97E-07	PSME_42829	uncharacterized protein
45°C	JCF7190000035918	seq-rs15169-DF	631159	G	A	0.09	3.99	1.14E-07	PSME_42829	uncharacterized protein

Figure S1. Heatmap showing correlations among environmental variables for all 110 Douglas-fir families. Color legend indicates the Pearson's correlation value (r). Crosses indicate no significant correlation between traits.

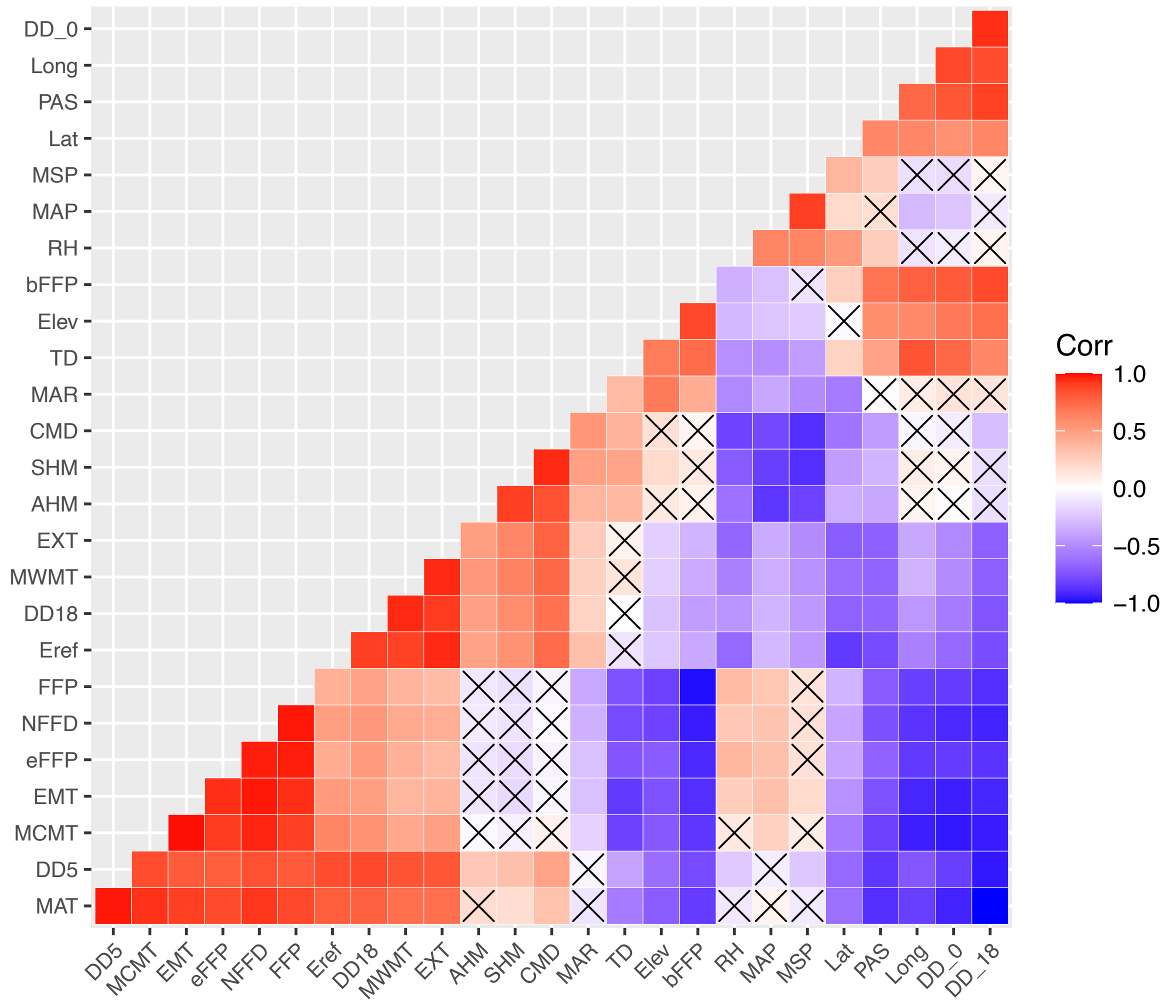

Figure S2. Heatmap showing correlations among environmental variables and heat tolerance at different temperatures, carbon isotope discrimination, nitrogen content, and growth traits for all Douglas-fir families. Color legend indicates the Pearson's correlation value (r). Crosses indicate no significant correlation between traits.

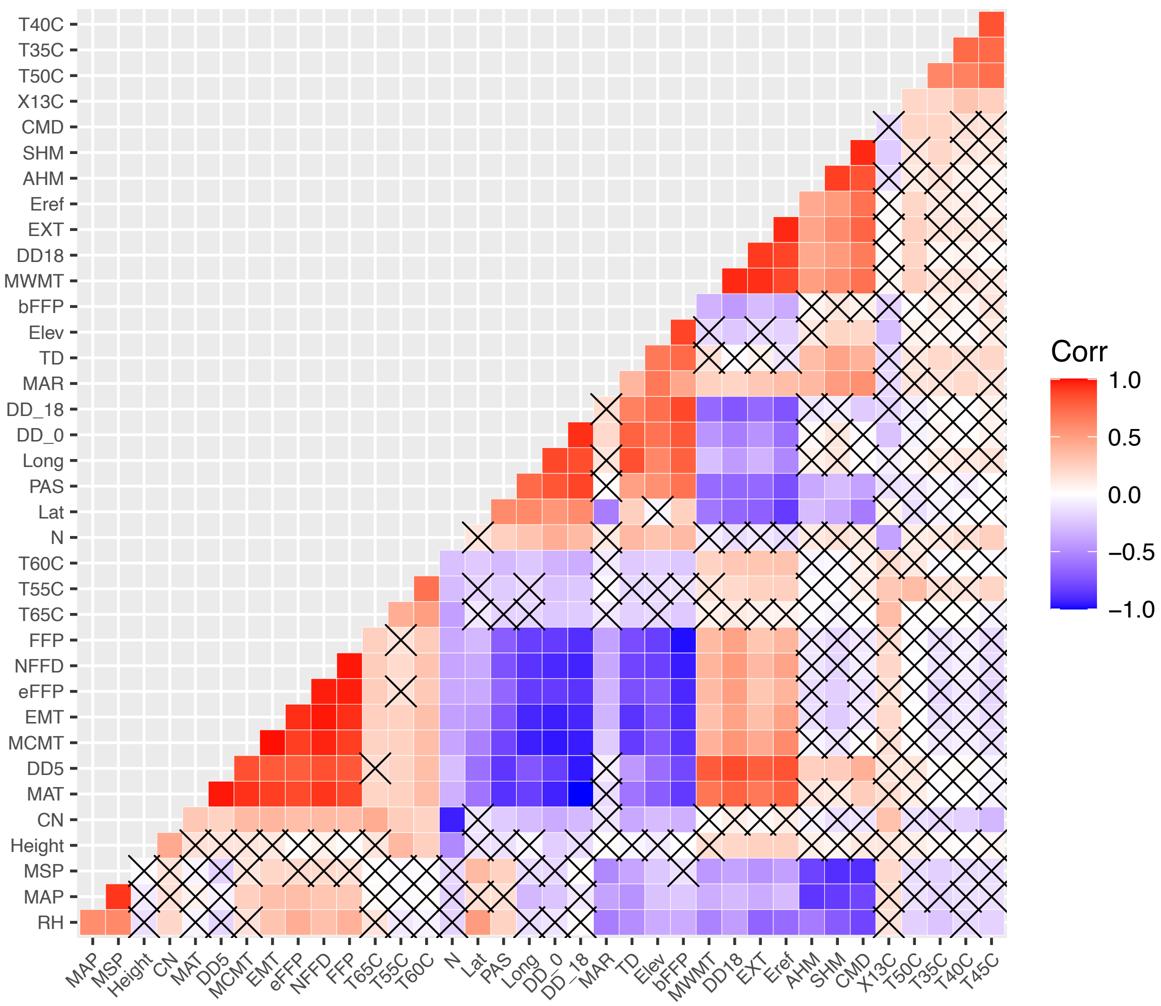

Figure S3. Principal components analysis (PCA) based on 14,980 SNP markers shows genetic differences between coastal variety (orange) and hybrid (blue) families in the dataset. Each family contains between 5 and 30 individuals.

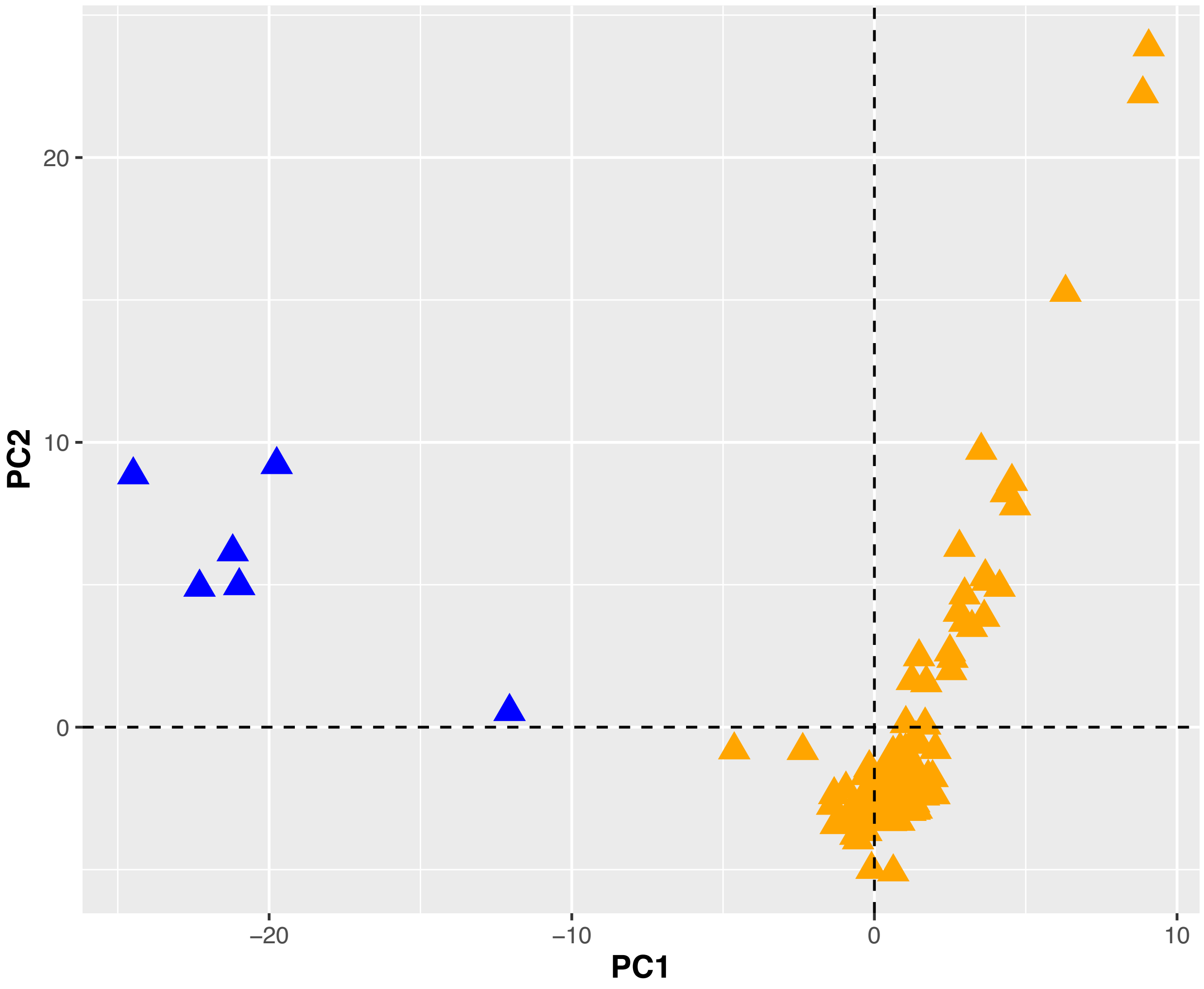

Figure S4. Ancestry estimates suggest the presence of intervarietal hybrids in this study. Mother trees of all families were analyzed as part of larger dataset of 464 individuals including coastal, interior and hybrids with 14,980 SNP markers in fastSTRUCTURE for K=2 (unpublished data). Ancestry barplot showed here only contains the mother trees of individuals included in this study. Intervarietal hybrids show a combination of coastal (orange bars) and interior (blue bars) ancestry, whereas pure coastal individuals show more than 80% ancestry from the coastal variety (orange bars).

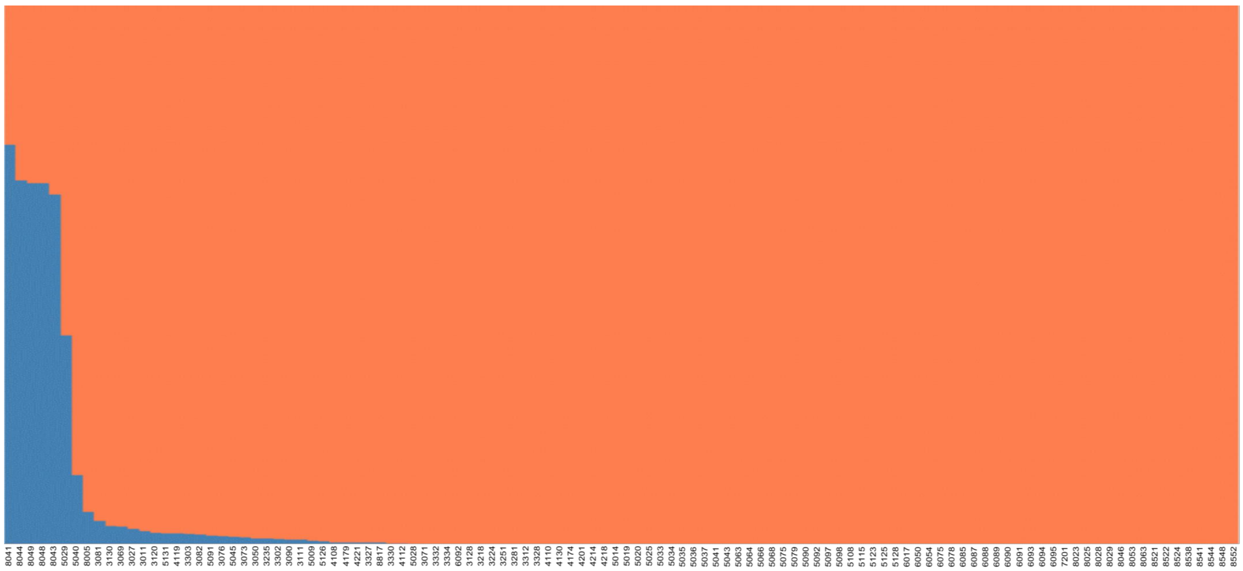

Supplement: plad008_suppl_Supplementary_Material [file plad008_suppl_supplementary_material.pdf]
